# Third-generation EGFR-TKI plus pemetrexed-platinum chemotherapy *versus* TKI monotherapy as first-line treatment for EGFR-mutant lung adenocarcinoma with brain parenchymal metastases

**DOI:** 10.3389/fphar.2026.1959801

**Published:** 2026-10-07

**Authors:** Jianxi Zhou, Yinghao Wang, Kang Wen, Chun Huang, Tingting Qin, Yunchuan Sun, Peng Chen

**Affiliations:** 1 Department of Head, Neck and Thoracic Oncology, Cangzhou Hospital of Integrated Traditional Chinese and Western Medicine-Hebei, Cangzhou, Hebei Province, China; 2 Key Laboratory of Cancer Prevention and Therapy, Tianjin Medical University Cancer Institute and Hospital, National Clinical Research Center for Cancer, Tianjin, China; 3 Department of Thoracic Oncology, Tianjin Lung Cancer Center, Tianjin’s Clinical Research Center for Cancer, Tianjin Cancer Institute & Hospital, Tianjin Medical University, Tianjin, China; 4 Department of Urology, The Fifth People’s Hospital of Hengshui City, Hengshui, Heibei Province, China

**Keywords:** brain parenchymal metastases, EGFR mutation, intracranial progression-free survival, lung adenocarcinoma, pemetrexed-platinum chemotherapy, third-generation EGFR-TKI

## Abstract

**Objective:**

To evaluate the efficacy and safety of first-line third-generation EGFR-tyrosine kinase inhibitor (EGFR-TKI) combined with pemetrexed-platinum chemotherapy in patients with EGFR-mutant advanced lung adenocarcinoma and brain parenchymal metastases.

**Methods:**

A bicenter retrospective cohort study was conducted. A total of 150 eligible patients were enrolled and balanced by 1:1 propensity score matching. Tumor response, survival outcomes, and treatment-related adverse events were compared between groups.

**Results:**

After matching, 60 patients were assigned to each group. The intracranial objective response rate was 78.3% in the combination group *versus* 61.7% in the monotherapy group (P = 0.046). The median intracranial progression-free survival was 28.3 months in the combination group (hazard ratio = 0.536, 95% confidence interval: 0.350–0.820, P = 0.002). Systemic progression-free survival and overall survival were also significantly prolonged in the combination arm. Adverse events were predominantly chemotherapy-related toxicities and were generally manageable.

**Conclusion:**

First-line third-generation EGFR-TKI plus pemetrexed-platinum chemotherapy is associated with superior intracranial and systemic survival benefits in EGFR-mutant lung adenocarcinoma patients with brain parenchymal metastases, with a favorable safety profile. This study provides real-world evidence to support clinical treatment decision-making.

## 1 Introduction

Lung cancer remains the leading cause of cancer-related mortality worldwide. Non-small cell lung cancer (NSCLC), of which lung adenocarcinoma is the predominant histological subtype, accounts for approximately 85% of all lung cancer cases (Bray et al., 2024; Leiter et al., 2023). Sensitizing epidermal growth factor receptor (EGFR) mutations are the most common driver alteration in lung adenocarcinoma, reaching a frequency of 40%–50% in Asian populations, substantially higher than that in Western cohorts (da Cunha Santos et al., 2011; Ma and Li, 2025). Brain parenchymal metastases are among the most frequent sites of distant spread in EGFR-mutant NSCLC: 20%–30% of patients present with synchronous brain metastases at diagnosis, and the cumulative incidence throughout the disease course can reach 40%–50%, significantly exceeding that of EGFR wild-type tumors (Wang et al., 2025). These metastases impair neurological function and quality of life. Moreover, brain parenchymal metastases act as independent negative prognostic factors (Sereno et al., 2024). In the absence of effective systemic therapy, affected patients have a median overall survival (OS) of only approximately 7 months (Pellerino et al., 2021).

Third-generation EGFR-TKIs, owing to their superior blood-brain barrier penetration and potent intracranial antitumor activity, have gradually replaced first- and second-generation agents as the standard first-line regimen for advanced NSCLC harboring EGFR sensitizing mutations (Herrera-Juárez et al., 2023; Lee et al., 2026). The pivotal phase III FLAURA trial demonstrated that first-line osimertinib prolonged progression-free survival (PFS) and OS compared with first-generation EGFR-TKIs and reduced intracranial progression in the predefined subgroup with central nervous system (CNS) metastases (Soria et al., 2018); domestic third-generation agents, including aumolertinib and furmonertinib, have subsequently confirmed comparable survival and intracranial benefits (Shi et al., 2022; Lu et al., 2022). Nevertheless, acquired resistance remains inevitable, and intracranial progression is a predominant mode of treatment failure. In patients presenting with multiple symptomatic brain metastases at baseline, TKI monotherapy provides only limited durable intracranial control, with a median intracranial progression-free survival (iPFS) of merely 15–20 months, underscoring the need for more effective first-line strategies (Passaro et al., 2021).

EGFR-TKI combined with chemotherapy has therefore been increasingly explored to delay resistance and strengthen intracranial and systemic control; the pemetrexed-platinum regimen is the standard chemotherapy backbone for advanced non-squamous NSCLC, and studies of first- and second-generation TKIs have confirmed longer PFS with combination therapy, together with a trend toward improved intracranial outcomes in patients with baseline brain metastases (Wu et al., 2021; Su et al., 2026). In the third-generation era, the phase III FLAURA2 trial established that adding pemetrexed-platinum chemotherapy to osimertinib improved both PFS and OS, and its exploratory subgroup with baseline CNS metastases provided signals of enhanced intracranial control (Gray et al., 2026; Planchard et al., 2023). Collectively, available evidence suggests that, among first-line options, third-generation EGFR-TKI combined with chemotherapy may deliver the greatest iPFS and OS benefits for patients with brain metastases (Jänne et al., 2026; Hou et al., 2023; Jia et al., 2024).

Despite these encouraging signals, current evidence remains limited. First, brain-metastasis subgroup analyses of pivotal phase III trials are exploratory, with small samples and limited statistical power because they were not designed specifically for cohorts with brain parenchymal metastases. Second, detailed data on endpoints critical for clinical decision-making, such as patterns of first intracranial progression and efficacy stratified by symptomatic brain metastasis status, are frequently lacking. Third, stringent trial eligibility criteria generate study populations that differ from the heterogeneous real-world cohort of patients with brain metastases, restricting generalizability. Consequently, large-scale real-world studies specifically comparing first-line third-generation EGFR-TKI plus pemetrexed-platinum chemotherapy with TKI monotherapy in EGFR-mutant lung adenocarcinoma with synchronous brain parenchymal metastases remain scarce.

To address this gap, we conducted a bicenter retrospective cohort study enrolling patients with advanced EGFR-sensitizing mutant lung adenocarcinoma and baseline brain parenchymal metastases who received first-line third-generation EGFR-TKI combined with pemetrexed-platinum chemotherapy or third-generation EGFR-TKI monotherapy. We systematically compared intracranial and systemic tumor responses, survival outcomes, patterns of intracranial progression, and treatment safety, and identified independent prognostic factors. As this is a retrospective cohort study, the inherent biases of observational research may persist even after propensity score matching (PSM); accordingly, the present study aims to generate real-world evidence rather than provide confirmative clinical recommendations, and its findings should inform, rather than replace, individualized first-line therapeutic decision-making.

## 2 Materials and methods

### 2.1 Study design and participants

This bicenter, retrospective observational cohort study recruited patients from Cangzhou Hospital of Integrated Traditional Chinese and Western Medicine and Tianjin Medical University Cancer Institute and Hospital. The study protocol was reviewed and approved by the institutional ethics committees of both participating centers. Owing to the retrospective nature of this investigation, informed consent was waived. All procedures complied with the ethical principles outlined in the Declaration of Helsinki.

We consecutively screened 406 patients diagnosed with advanced EGFR-mutant lung adenocarcinoma between January 2022 and December 2025 (217 patients from Center 1 and 189 patients from Center 2). After sequential screening according to predefined inclusion and exclusion criteria, 256 patients were excluded. Exclusion grounds included prior systemic antitumor therapy or cranial local treatment, absence of brain parenchymal metastases confirmed by baseline contrast-enhanced brain magnetic resonance imaging (MRI), predominant leptomeningeal metastases, non-adenocarcinoma histology, missing key clinical data, and follow-up duration shorter than 1 month. Ultimately, 150 patients fulfilling all eligibility criteria constituted the full analysis set, including 64 patients receiving third-generation EGFR-TKI plus pemetrexed-platinum chemotherapy and 86 patients treated with third-generation EGFR-TKI monotherapy. The patient screening flowchart is presented in Figure 1. To balance baseline confounding variables and minimize selection bias, 1:1 nearest-neighbor propensity score matching (PSM) was performed with a caliper of 0.05. Thirty unmatched patients were excluded after matching (4 from the combination group and 26 from the monotherapy group). The post-matching primary analysis set comprised 120 patients, evenly divided into two groups of 60 individuals each. All primary statistical analyses were performed on the matched population.

**FIGURE 1:**
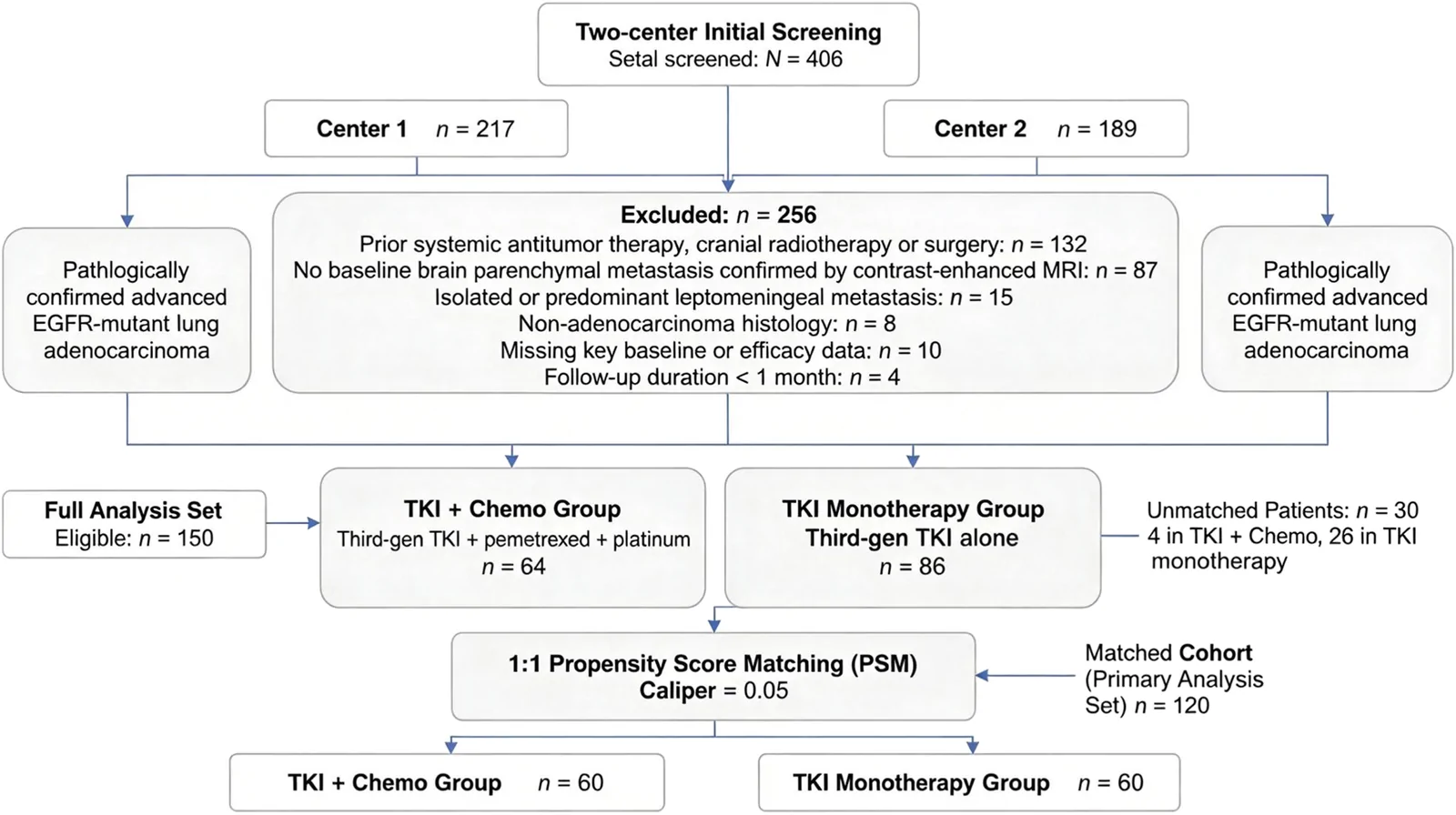
CONSORT flowchart of patient enrollment and propensity score matching. A total of 406 patients with advanced EGFR-mutant lung adenocarcinoma from two centers were screened between January 2022 and December 2025. After exclusion of ineligible cases, 150 patients comprised the full analysis set. 1:1 nearest-neighbor propensity score matching (caliper = 0.05) yielded 120 patients (60 per arm) for the primary matched analysis.

Inclusion criteria: ① Histopathologically confirmed lung adenocarcinoma with molecular validation of EGFR sensitizing mutations (exon 19 deletion or exon 21 L858R point mutation); ② Brain parenchymal metastases verified by baseline contrast-enhanced brain MRI; ③ Stage IV disease at initial diagnosis without prior antitumor treatment; ④ First-line treatment consisting of either single-agent third-generation EGFR-TKI or third-generation EGFR-TKI combined with pemetrexed-platinum chemotherapy; ⑤ Complete evaluable baseline clinical, imaging and follow-up data.

Exclusion criteria: ① Concurrent known alternative driver gene alterations including ALK and ROS1 rearrangements; ② Isolated leptomeningeal metastases without parenchymal brain lesions; ③ Prior cranial radiotherapy, chemotherapy, or other antitumor interventions administered before baseline systemic therapy; ④ History of other primary malignant neoplasms; ⑤ Incomplete clinical, pathological or follow-up documentation.

### 2.2 Treatment regimens

All therapeutic strategies were determined by attending clinicians based on patients’ performance status, disease characteristics and individual preferences, consistent with recommendations from the National Comprehensive Cancer Network (NCCN) NSCLC Clinical Practice Guidelines and Chinese Society of Clinical Oncology (CSCO) NSCLC Guidelines.

Monotherapy group: Patients received oral third-generation EGFR-TKI, including osimertinib (80 mg once daily), aumolertinib (110 mg once daily), or furmonertinib (80 mg once daily). Treatment continued until disease progression, intolerable adverse events, or voluntary treatment discontinuation.

Combination group: Patients received third-generation EGFR-TKI (identical dosing schedule as the monotherapy group) plus concurrent pemetrexed-platinum chemotherapy. The chemotherapy regimen consisted of pemetrexed 500 mg/m^2^ intravenously on Day 1 combined with either cisplatin 75 mg/m^2^ intravenously on Day 1 or carboplatin at an area under the curve (AUC) of 5 intravenously on Day 1, administered every 21 days as one cycle. Induction chemotherapy was delivered for 4–6 cycles. Following completion of induction chemotherapy, patients maintained third-generation EGFR-TKI plus single-agent pemetrexed until disease progression, unacceptable toxicity, or treatment termination. Supportive care including folic acid, vitamin B12, and 5-hydroxytryptamine receptor antagonists was routinely administered during chemotherapy.

### 2.3 Efficacy assessment and study endpoints

At baseline, all patients underwent contrast-enhanced brain MRI, contrast-enhanced chest and abdominal computed tomography (CT), and whole-body bone scintigraphy to quantify baseline tumor burden and characterize brain metastases. Imaging reassessment with contrast-enhanced brain MRI and systemic scans was conducted every 6–8 weeks during treatment, and regular follow-up imaging continued after disease progression. Tumor response was independently evaluated for intracranial and systemic lesions according to Response Evaluation Criteria in Solid Tumors version 1.1 (RECIST 1.1) (Schwartz et al., 2016). Responses were categorized as complete response (CR), partial response (PR), stable disease (SD), and progressive disease (PD). The primary endpoint was iPFS, defined as the interval from initiation of first-line therapy to the first documented intracranial progression or all-cause death, whichever occurred earlier. Patients without intracranial progression who remained alive were censored at the date of last follow-up. Secondary endpoints encompassed: ① PFS: time from treatment initiation to systemic disease progression or all-cause death; ② OS: time from treatment initiation to all-cause death; ③ Intracranial objective response rate (iORR) and intracranial disease control rate (iDCR): the proportions of patients achieving CR + PR and CR + PR + SD for intracranial lesions, respectively; ④ Systemic objective response rate (ORR) and systemic disease control rate (DCR); ⑤ Patterns of first intracranial progression, classified into three categories: progression of existing target lesions only, new brain metastatic lesions only, concurrent progression of existing lesions plus new brain metastases; ⑥ Incidence and grade distribution of treatment-related adverse events (TRAEs); ⑦ Differences in intracranial survival benefits across clinical subgroups; ⑧ Independent prognostic factors for iPFS, PFS and OS.

Adverse events were graded and documented per the National Cancer Institute Common Terminology Criteria for Adverse Events version 5.0 (NCI-CTCAE v5.0) (United States Department of Health and Human Services, 2017).

### 2.4 Follow-up

Comprehensive follow-up was implemented via medical record review, outpatient system tracking, and regular telephone contact. During treatment, efficacy evaluation was performed every 6–8 weeks, incorporating contrast-enhanced brain MRI, chest and abdominal contrast-enhanced CT, laboratory monitoring of blood routine, liver and renal function, and tumor markers. Whole-body bone scintigraphy or PET-CT was arranged as clinically indicated. After disease progression or treatment cessation, follow-up visits were scheduled every 3 months within the initial 2 years and every 6 months thereafter. Follow-up information captured survival status, intracranial and systemic progression events, subsequent treatment modifications, and resolution of treatment-related adverse events. Patients presenting with new or worsening neurological symptoms received timely imaging evaluation. The cutoff date for follow-up was 1 July 2026. The reverse Kaplan-Meier method was applied to calculate median follow-up duration. For the full analysis set, the median follow-up time was 32.4 months (interquartile range [IQR]: 24.7–37.1 months).

### 2.5 Statistical analysis

Propensity score matching (PSM) was performed to implement 1:1 nearest-neighbor matching with a caliper of 0.05 to balance baseline covariates and mitigate selection bias. Covariates incorporated into the matching model included age, sex, Eastern Cooperative Oncology Group performance status (ECOG PS), smoking history, EGFR mutation subtype, number of extracranial metastatic sites, presence of bone metastasis, presence of liver metastasis, number of brain metastases, maximum diameter of brain lesions, symptomatic brain metastasis status, peritumoral edema severity, Graded Prognostic Assessment (GPA) score, and type of third-generation EGFR-TKI. Standardized mean difference (SMD) was used to assess post-matching baseline balance; an SMD <0.1 indicated satisfactory covariate balance. A total of 120 matched patients (60 per group) constituted the primary analysis population.

Normally distributed continuous variables were expressed as mean ± standard deviation and compared using independent samples t-test. Non-normal continuous variables were presented as median (IQR), with intergroup comparisons performed via the Mann-Whitney U test. Categorical variables were summarized as frequencies and percentages, compared using the χ^2^ test or Fisher’s exact test where appropriate. Survival curves were generated by the Kaplan-Meier method, and between-group survival differences were tested with the Log-rank test. Cox proportional hazards regression models were adopted to calculate hazard ratios (HR) and corresponding 95% confidence intervals (CI). The proportional hazards (PH) assumption underlying every Cox model was formally examined using scaled Schoenfeld residual (Grambsch–Therneau) tests, evaluated both globally and for each covariate; a two-sided P value <0.05 was considered indicative of a departure from proportionality. Prespecified subgroup analyses of iPFS were conducted across eight clinicopathological features, and interaction tests were applied to evaluate heterogeneity of treatment benefit across subgroups.

Univariate Cox proportional hazards models were used to screen candidate prognostic factors associated with iPFS, PFS and OS. Variables with P < 0.2 in univariate analysis were entered into multivariate Cox regression models, with stepwise selection utilized to identify independent prognostic determinants. All statistical analyses were executed using SPSS 26.0 (IBM Corp., Armonk, NY, USA) and R 4.3.1 (R Foundation for Statistical Computing, Vienna, Austria). Two-tailed tests were applied throughout, and a P value <0.05 was defined as statistically significant.

## 3 Results

### 3.1 Baseline clinicopathological characteristics of patients

Baseline characteristics before PSM are summarized in Table 1. The combination group exhibited marginally more favorable baseline prognostic profiles, including a higher proportion of oligometastatic brain disease and lower rates of symptomatic brain metastases and moderate-to-severe peritumoral edema, though no differences reached statistical significance (all P > 0.05). After PSM, the two groups achieved balanced distributions across age, sex, ECOG PS, smoking history, EGFR mutation subtype, extracranial metastatic burden, bone/liver metastasis status, number and maximum diameter of brain metastases, symptomatic status, peritumoral edema severity, GPA score, and type of third-generation TKI. Apart from symptomatic brain metastasis (SMD = 0.108), all other covariates yielded SMD values below 0.1, indicating adequate overall matching and satisfying baseline balance requirements for subsequent intergroup comparisons (Table 1).

**TABLE 1:** Baseline clinicopathological characteristics of patients before and after propensity score matching.

Characteristic	Full cohort before PSM (n = 150)	Monotherapy group (n = 86)	P	Matched cohort after PSM (n = 120)	Monotherapy group (n = 60)	P	SMD
	Combination group (n = 64)			Combination group (n = 60)			
Age, years, median (IQR)	58 (51–66)	61 (53–69)	0.092	59 (52–67)	60 (52–68)	0.784	0.031
Sex, n (%)	​	​	0.633	​	​	0.855	0.034
Male	28 (43.8)	41 (47.7)	​	27 (45.0)	28 (46.7)	​	​
Female	36 (56.2)	45 (52.3)	​	33 (55.0)	32 (53.3)	​	​
ECOG PS, n (%)	​	​	0.212	​	​	0.793	0.045
0–1	55 (85.9)	67 (77.9)	​	52 (86.7)	51 (85.0)	​	​
≥2	9 (14.1)	19 (22.1)	​	8 (13.3)	9 (15.0)	​	​
Smoking history, n (%)	​	​	0.615	​	​	0.849	0.035
Never-smoker	42 (65.6)	53 (61.6)	​	38 (63.3)	39 (65.0)	​	​
Former smoker	22 (34.4)	33 (38.4)	​	22 (36.7)	21 (35.0)	​	​
EGFR mutation subtype, n (%)	​	​	0.564	​	​	0.853	0.033
Exon 19 deletion	38 (59.4)	47 (54.7)	​	35 (58.3)	34 (56.7)	​	​
L858R point mutation	26 (40.6)	39 (45.3)	​	25 (41.7)	26 (43.3)	​	​
Number of extracranial metastatic sites, n (%)	​	​	0.234	​	​	0.854	0.034
0–1	39 (60.9)	44 (51.2)	​	33 (55.0)	34 (56.7)	​	​
≥2	25 (39.1)	42 (48.8)	​	27 (45.0)	26 (43.3)	​	​
Bone metastasis, n (%)	​	​	0.381	​	​	0.699	0.070
Yes	23 (35.9)	37 (43.0)	​	19 (31.7)	21 (35.0)	​	​
No	41 (64.1)	49 (57.0)	​	41 (68.3)	39 (65.0)	​	​
Liver metastasis, n (%)	​	​	0.617	​	​	0.810	0.042
Yes	12 (18.8)	19 (22.1)	​	11 (18.3)	10 (16.7)	​	​
No	52 (81.2)	67 (77.9)	​	49 (81.7)	50 (83.3)	​	​
Number of brain metastases, n (%)	​	​	0.194	​	​	1.000	0.000
1–3 (oligometastases)	41 (64.1)	46 (53.5)	​	36 (60.0)	36 (60.0)	​	​
≥4 (multiple metastases)	23 (35.9)	40 (46.5)	​	24 (40.0)	24 (40.0)	​	​
Maximum diameter of brain metastases, cm, median (IQR)	1.8 (1.2–2.5)	2.0 (1.3–2.8)	0.087	1.9 (1.2–2.6)	1.9 (1.3–2.7)	0.815	0.028
Symptomatic brain metastasis, n (%)	​	​	0.169	​	​	0.540	0.108
Yes	17 (26.6)	32 (37.2)	​	15 (25.0)	18 (30.0)	​	​
No	47 (73.4)	54 (62.8)	​	45 (75.0)	42 (70.0)	​	​
Moderate-to-severe peritumoral edema, n (%)	​	​	0.465	​	​	0.841	0.037
Yes	18 (28.1)	29 (33.7)	​	18 (30.0)	17 (28.3)	​	​
No	46 (71.9)	57 (66.3)	​	42 (70.0)	43 (71.7)	​	​
GPA score, n (%)	​	​	0.465	​	​	1.000	0.000
0–1.5	18 (28.1)	29 (33.7)	​	16 (26.7)	16 (26.7)	​	​
2–4	46 (71.9)	57 (66.3)	​	44 (73.3)	44 (73.3)	​	​
Type of third-generation TKI, n (%)	​	​	0.708	​	​	0.843	0.037
Osimertinib	45 (70.3)	58 (67.4)	​	41 (68.3)	42 (70.0)	​	​
Aumolertinib/Furmonertinib	19 (29.7)	28 (32.6)	​	19 (31.7)	18 (30.0)	​	​

Data are presented as median (IQR) for continuous variables and n (%) for categorical variables. P values were calculated via χ^2^ test, Fisher’s exact test or Mann–Whitney U test as appropriate. 1:1 nearest-neighbor PSM, was performed with a caliper width of 0.05. SMD <0.1 indicates adequate covariate balance.

Abbreviations: PSM, propensity score matching; SMD, standardized mean difference; ECOG PS, eastern cooperative oncology group performance status; GPA, graded prognostic assessment; IQR, interquartile range.

### 3.2 Intracranial and systemic tumor responses

Best overall tumor responses for the matched cohort are outlined in Table 2. For intracranial lesions: 6 patients achieved CR, 41 achieved PR, 11 achieved SD, and 2 experienced PD in the combination group; 3 achieved CR, 34 achieved PR, 17 achieved SD, and 6 experienced PD in the monotherapy group. The iORR was significantly higher in the combination group (78.3%) *versus* the monotherapy group (61.7%, P = 0.046). The iDCR reached 96.7% and 90.0% in the two groups, respectively, without statistically significant difference (P = 0.272).

**TABLE 2:** Intracranial and systemic best overall tumor response after propensity score matching.

Response endpoint	Combination group (n = 60)	Monotherapy group (n = 60)	P
Intracranial response			
Complete response (CR), n (%)	6 (10.0)	3 (5.0)	–
Partial response (PR), n (%)	41 (68.3)	34 (56.7)	–
Stable disease (SD), n (%)	11 (18.3)	17 (28.3)	–
Progressive disease (PD), n (%)	2 (3.3)	6 (10.0)	–
Intracranial objective response rate (iORR), n (%)	47 (78.3)	37 (61.7)	0.046
Intracranial disease control rate (iDCR), n (%)	58 (96.7)	54 (90.0)	0.272
Systemic response			
Complete response (CR), n (%)	4 (6.7)	2 (3.3)	–
Partial response (PR), n (%)	45 (75.0)	37 (61.7)	–
Stable disease (SD), n (%)	10 (16.7)	16 (26.7)	–
Progressive disease (PD), n (%)	1 (1.7)	5 (8.3)	–
Systemic objective response rate (ORR), n (%)	49 (81.7)	39 (65.0)	0.039
Systemic disease control rate (DCR), n (%)	59 (98.3)	55 (91.7)	0.207

Tumor response was assessed per RECIST v1.1. iORR, was defined as CR + PR; iDCR, was defined as CR + PR + SD. P values were derived from χ^2^ test or Fisher’s exact test.

Abbreviations: CR, complete response; PR, partial response; SD, stable disease; PD, progressive disease; iORR, intracranial objective response rate; iDCR, intracranial disease control rate; ORR, objective response rate; DCR, disease control rate.

For systemic lesions: 4 patients achieved CR, 45 achieved PR, 10 achieved SD, and 1 experienced PD in the combination group; 2 achieved CR, 37 achieved PR, 16 achieved SD, and 5 experienced PD in the monotherapy group. The systemic ORR was 81.7% in the combination group *versus* 65.0% in the monotherapy group (P = 0.039). Systemic DCR was 98.3% and 91.7%, respectively, with no significant intergroup difference (P = 0.207).

### 3.3 Survival outcome analyses

Survival curves for the matched population are displayed in Figure 2. Across the full analysis set, the median iPFS was 18.9 months, median PFS was 17.2 months, and median OS was 29.5 months (Figure 2A). The proportional hazards assumption was satisfied for all Cox regression models. For the univariable treatment-arm models, scaled Schoenfeld residual tests showed no departure from proportionality for iPFS (χ^2^ = 0.84, df = 1, P = 0.359), systemic PFS (χ^2^ = 1.12, df = 1, P = 0.290), or OS (χ^2^ = 0.67, df = 1, P = 0.413). The global tests for the multivariable iPFS, PFS and OS models were likewise non-significant (global χ^2^ = 6.93, P = 0.436; χ^2^ = 7.58, P = 0.371; and χ^2^ = 8.21, P = 0.314, respectively; degrees of freedom = 7 for each), and every covariate-specific test returned P > 0.05 (Supplementary Table S1). The hazard ratios reported below are therefore derived from models that met the PH assumption.

**FIGURE 2:**
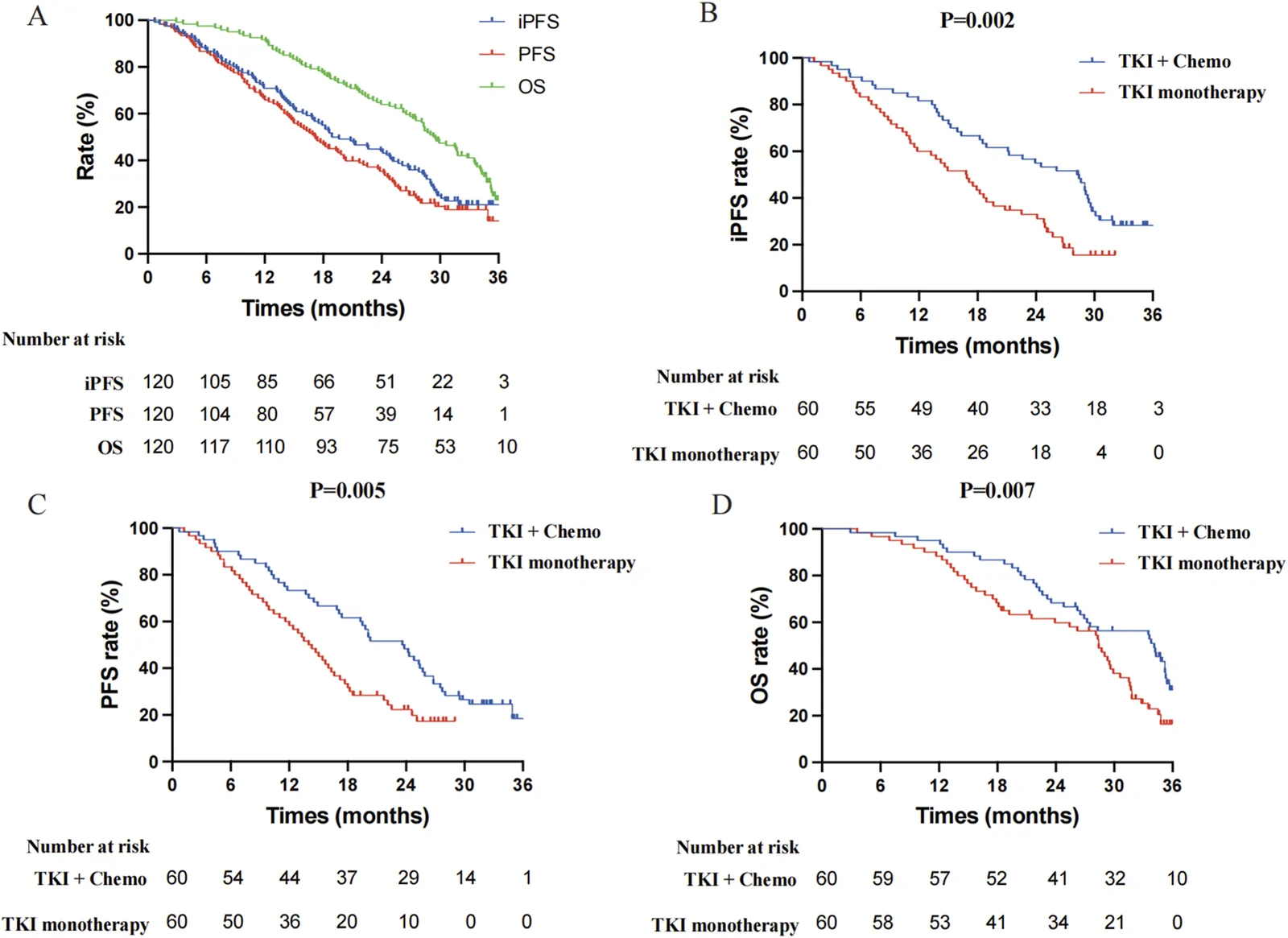
Kaplan–Meier survival curves. **(A)** Shown are median survival outcomes of the full cohort: iPFS 18.9 months, PFS 17.2 months, OS 29.5 months. **(B)** iPFS in the propensity score-matched cohort (combination therapy vs. monotherapy). **(C)** Systemic PFS in the propensity score-matched cohort. **(D)** OS in the propensity score-matched cohort. Survival differences were compared via Log-rank test. Hazard ratios (HRs) and 95% confidence intervals (CIs) were calculated using Cox proportional hazards regression.

For intracranial progression-free survival: the median iPFS was 28.3 months in the combination group and 16.9 months in the monotherapy group. Combination therapy reduced the risk of intracranial progression by 46.4% (HR = 0.536, 95%CI: 0.350–0.820, Log-rank P = 0.002). The 6-month iPFS rates were 91.7% *versus* 83.3%; the 12-month iPFS rates were 81.7% *versus* 60.0%; the 24-month iPFS rates were 55.0% *versus* 30.0% (Figure 2B). For systemic progression-free survival: median PFS was 23.7 months (combination) *versus* 14.2 months (monotherapy). Combination therapy reduced the risk of systemic progression or death by 42.7% (HR = 0.573, 95%CI: 0.377–0.870, Log-rank P = 0.005). The 6-month PFS rates were 90.0% *versus* 83.3%; the 12-month PFS rates were 73.3% *versus* 60.0%; the 24-month PFS rates were 48.3% *versus* 16.7% (Figure 2C). For overall survival: median OS was 34.1 months in the combination group and 28.4 months in the monotherapy group. Combination therapy reduced all-cause mortality risk by 42.6% (HR = 0.574, 95%CI: 0.372–0.883, Log-rank P = 0.007). The 6-month OS rates were 98.3% *versus* 96.7%; the 12-month OS rates were 95.0% *versus* 88.3%; the 24-month OS rates were 68.3% *versus* 56.7%; the 30-month OS rates were 53.3% *versus* 35.0% (Figure 2D).

### 3.4 Subgroup analyses of intracranial PFS

Prespecified subgroup analyses of iPFS across eight clinicopathological variables are illustrated in Figure 3. A consistent trend of improved intracranial survival with combination therapy was observed across all subgroups. Interaction tests revealed no statistically significant between-subgroup heterogeneity (all interaction P > 0.05), indicating that the intracranial survival benefit conferred by combination therapy remained consistent regardless of baseline age, sex, ECOG PS, EGFR mutation subtype, number of brain metastases, symptomatic brain metastasis status, extracranial metastatic burden, and type of third-generation TKI. More pronounced statistical trends favoring combination therapy were observed in subgroups aged ≤65 years, female patients, patients with ECOG PS 0–1, exon 19 deletion carriers, patients with 1–3 brain metastases, asymptomatic brain metastases, 0–1 extracranial metastatic sites, and patients receiving osimertinib. Nevertheless, no significant interaction effects were identified, and no distinct population with superior differential benefit was determined.

**FIGURE 3:**
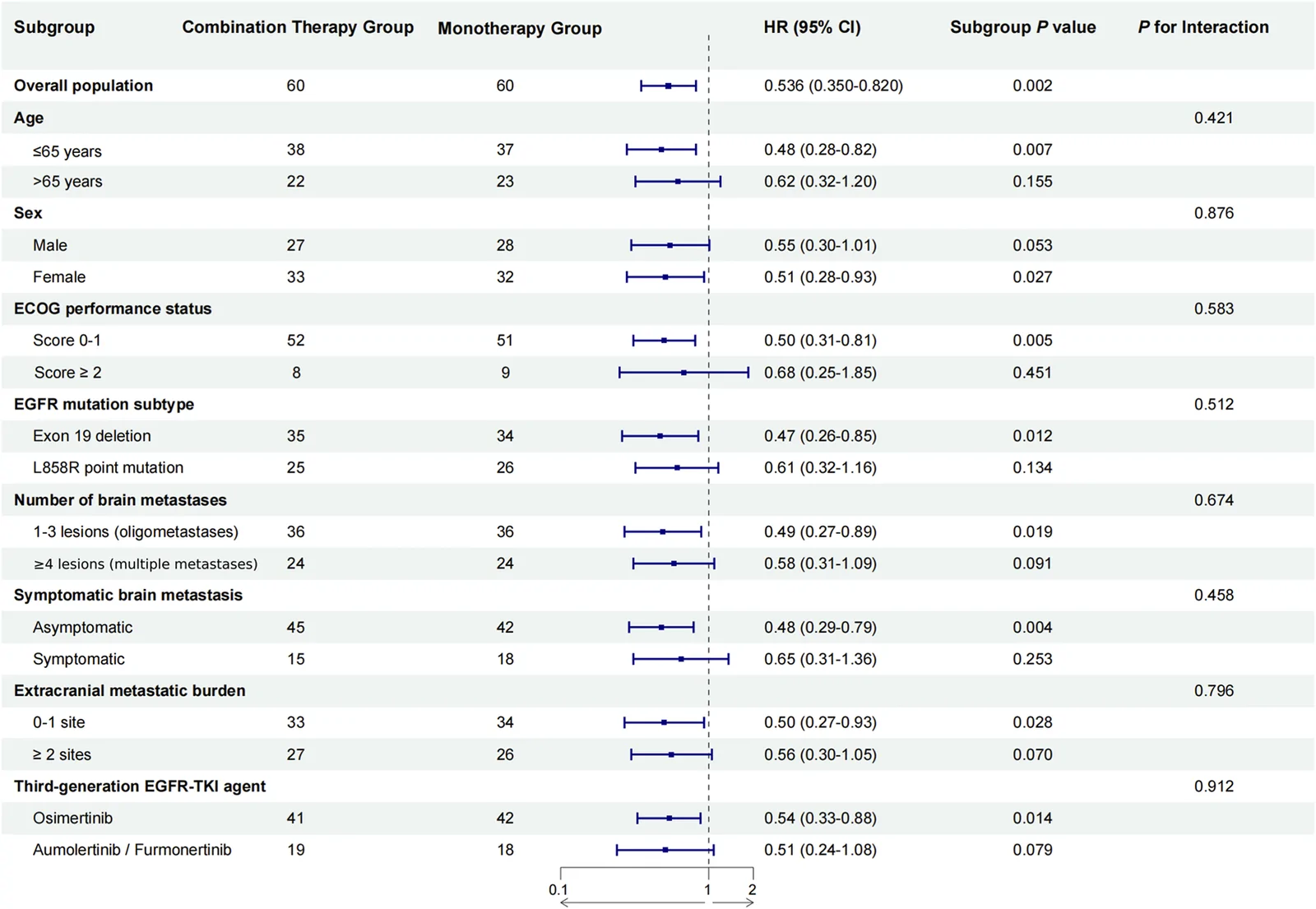
Forest plot of prespecified subgroup analyses for intracranial progression-free survival. HRs and 95% CIs for iPFS are presented for each clinical subgroup in the matched cohort. Interaction tests were performed to assess heterogeneity of treatment effect across subgroups.

### 3.5 Patterns of first intracranial progression

By the cutoff date of follow-up, intracranial progression occurred in 75 patients within the matched cohort, including 32 patients in the combination group and 43 patients in the monotherapy group. The distribution of first intracranial progression patterns is presented in Figure 4. In the combination group, progression confined to existing target lesions occurred in 17 patients (53.1%), isolated new brain metastases developed in 10 patients (31.3%), and concurrent progression of pre-existing lesions plus new metastases was documented in 5 patients (15.6%). Corresponding proportions in the monotherapy group were 18 (41.9%), 17 (39.5%), and 8 (18.6%). The overall distribution of progression patterns was not statistically different between groups (P = 0.624). Numerically, the combination group exhibited a lower proportion of new brain metastatic lesions and a higher proportion of progression limited to existing target lesions.

**FIGURE 4:**
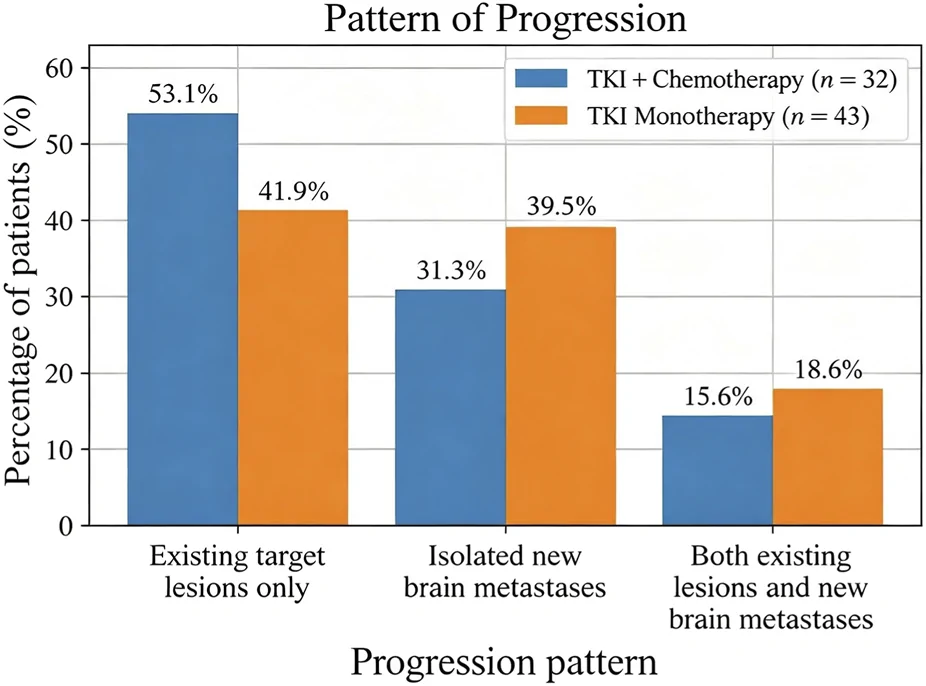
Patterns of first intracranial progression in the matched cohort. First intracranial progression was categorized into three patterns: progression of existing target lesions only, new brain metastatic lesions only, and concurrent progression of existing lesions plus new brain metastases. Between-group differences in distribution were tested using the χ^2^ test.

### 3.6 Safety analysis

Treatment-related adverse events for the matched population are summarized in Table 3. The incidence of any-grade TRAEs was 86.7% in the combination group *versus* 68.3% in the monotherapy group; ≥grade 3 TRAEs occurred in 30.0% *versus* 10.0% of patients, respectively. Both intergroup differences achieved statistical significance (P = 0.016 and P = 0.006).

**TABLE 3:** Treatment-related adverse events in the propensity score-matched cohort.

Adverse event	Combination group (n = 60) Any grade, n (%)	≥grade 3, n (%)	Monotherapy group (n = 60)	≥grade 3, n (%)	P (any grade)	P (≥grade 3)
			Any grade, n (%)			
Hematological toxicities						
Neutropenia	25 (41.7)	9 (15.0)	7 (11.7)	1 (1.7)	<0.001	0.017
Anemia	18 (30.0)	3 (5.0)	10 (16.7)	0 (0.0)	0.084	0.244
Thrombocytopenia	12 (20.0)	2 (3.3)	5 (8.3)	0 (0.0)	0.067	0.496
Gastrointestinal toxicities						
Nausea and vomiting	22 (36.7)	3 (5.0)	11 (18.3)	1 (1.7)	0.025	0.619
Diarrhea	16 (26.7)	2 (3.3)	14 (23.3)	1 (1.7)	0.673	1.000
Decreased appetite	19 (31.7)	2 (3.3)	10 (16.7)	0 (0.0)	0.055	0.496
Cutaneous and soft tissue toxicities						
Rash	14 (23.3)	1 (1.7)	13 (21.7)	1 (1.7)	0.827	1.000
Paronychia	9 (15.0)	0 (0.0)	8 (13.3)	0 (0.0)	0.793	–
Hepatic toxicity						
Elevated transaminases	13 (21.7)	2 (3.3)	8 (13.3)	1 (1.7)	0.230	1.000
CNS-specific adverse events						
Worsening peritumoral edema	8 (13.3)	2 (3.3)	6 (10.0)	2 (3.3)	0.570	1.000
Treatment-related headache	10 (16.7)	1 (1.7)	8 (13.3)	0 (0.0)	0.609	1.000
Other systemic toxicities						
Fatigue	20 (33.3)	2 (3.3)	13 (21.7)	1 (1.7)	0.152	1.000
Stomatitis	7 (11.7)	1 (1.7)	3 (5.0)	0 (0.0)	0.186	1.000
Overall adverse events						
Any-grade TRAE	52 (86.7)	–	41 (68.3)	–	0.016	–
≥grade 3 TRAE	18 (30.0)	–	6 (10.0)	–	0.006	–

Adverse events were graded according to NCI-CTCAE v5.0. P values compare event rates between the two groups using χ^2^ test or Fisher’s exact test.

Abbreviations: TRAE, treatment-related adverse event; CTCAE, common terminology criteria for adverse events.

Hematological toxicities constituted the predominant adverse event category in the combination group. Any-grade neutropenia occurred in 41.7% of patients, with grade ≥3 neutropenia observed in 15.0%, significantly higher than the monotherapy group (P < 0.05). Rates of anemia and thrombocytopenia were numerically higher in the combination group without reaching statistical significance. For gastrointestinal toxicity, any-grade nausea and vomiting were reported in 36.7% of patients receiving combination therapy compared with 18.3% in the monotherapy group (P = 0.025), though ≥ grade 3 events were rare, supporting acceptable overall tolerability. The incidence of diarrhea was comparable between groups. Rates of common TKI-associated toxicities including cutaneous adverse events (rash, paronychia) and elevated liver transaminases displayed no significant intergroup differences.

For CNS-specific adverse events, the incidence of worsening peritumoral edema was 13.3% and treatment-related headache occurred in 16.7% of patients in the combination group, with no significant disparities *versus* monotherapy. No treatment-related intracranial hemorrhage, seizure, or CNS death was recorded. All ≥ grade 3 adverse events resolved following dose modification or supportive management, and no treatment-related deaths occurred.

### 3.7 Prognostic factor analyses

Univariate and multivariate Cox proportional hazards regression analyses for iPFS are shown in Table 4. Univariate analysis demonstrated that treatment regimen, ECOG PS, number of brain metastases, and symptomatic brain metastasis were significantly associated with iPFS (all P < 0.05). Variables with P < 0.2 on univariate analysis were incorporated into multivariate models. After adjustment, combination therapy (HR = 0.52, 95%CI: 0.32–0.84, P = 0.008), ECOG PS 0–1 (HR = 0.52, 95%CI: 0.29–0.93, P = 0.026), and 1–3 brain metastases (HR = 0.53, 95%CI: 0.33–0.87, P = 0.011) emerged as independent protective factors for iPFS. Symptomatic brain metastasis represented an independent adverse prognostic factor (HR = 1.68, 95%CI: 1.02–2.77, P = 0.042).

**TABLE 4:** Independent prognostic factors for intracranial progression-free survival (iPFS) after propensity score matching, Cox proportional hazards model.

Variable	Comparison	Univariate analysis	P	Multivariate analysis	P
		HR (95% CI)		HR (95% CI)	
Treatment regimen	Combination vs. monotherapy	0.54 (0.34–0.85)	0.006	0.52 (0.32–0.84)	0.008
Age	>65 years vs. ≤ 65 years	1.24 (0.79–1.95)	0.342	1.18 (0.73–1.91)	0.495
ECOG PS	≥2 vs. 0–1	2.01 (1.16–3.48)	0.013	1.92 (1.08–3.41)	0.026
EGFR mutation subtype	Exon19del vs. L858R	0.71 (0.45–1.12)	0.141	0.69 (0.43–1.11)	0.126
Number of brain metastases	≥4 vs. 1–3	1.93 (1.22–3.05)	0.005	1.87 (1.15–3.04)	0.011
Symptomatic brain metastasis	Yes vs. No	1.78 (1.11–2.86)	0.017	1.68 (1.02–2.77)	0.042
Number of extracranial metastatic sites	≥2 vs. 0–1	1.42 (0.90–2.24)	0.132	1.35 (0.84–2.18)	0.213

Variables with P < 0.2 in univariate analysis were entered into the multivariate stepwise Cox model. The second category in each comparison served as the reference group.

Abbreviations: HR, hazard ratio; CI, confidence interval; ECOG PS, eastern cooperative oncology group performance status.

Multivariate prognostic models for systemic PFS and OS are listed in Table 5. For systemic PFS, combination therapy, ECOG PS 0–1, and 1–3 brain metastases were independent favorable predictors (all P < 0.05). For OS, independent protective factors included combination therapy (HR = 0.58, 95%CI: 0.37–0.91, P = 0.017), ECOG PS 0–1, and fewer than two extracranial metastatic sites. Symptomatic brain metastasis independently predicted inferior OS (HR = 1.82, 95%CI: 1.05–3.15, P = 0.033).

**TABLE 5:** Independent prognostic factors for systemic PFS and OS after propensity score matching.

Variable	Comparison	Systemic PFS	P	Overall survival (OS)	P
		HR (95% CI)		HR (95% CI)	
Treatment regimen	Combination vs. monotherapy	0.56 (0.36–0.87)	0.007	0.58 (0.37–0.91)	0.017
Age	>65 years vs. ≤65 years	1.19 (0.76–1.87)	0.447	1.31 (0.77–2.23)	0.320
ECOG PS	≥2 vs. 0–1	1.85 (1.07–3.20)	0.028	2.09 (1.12–3.90)	0.021
EGFR mutation subtype	Exon19del vs. L858R	0.73 (0.47–1.14)	0.165	0.76 (0.44–1.31)	0.322
Number of brain metastases	≥4 vs. 1–3	1.76 (1.12–2.77)	0.014	1.64 (0.95–2.83)	0.076
Symptomatic brain metastasis	Yes vs. No	1.57 (0.99–2.49)	0.054	1.82 (1.05–3.15)	0.033
Number of extracranial metastatic sites	≥2 vs. 0–1	1.47 (0.93–2.31)	0.099	1.75 (1.01–3.02)	0.046

Multivariate stepwise Cox regression was applied. The second category in each comparison served as the reference group.

Abbreviations: HR, hazard ratio; CI, confidence interval; PFS, progression-free survival; OS, overall survival; ECOG PS, eastern cooperative oncology group performance status.

## 4 Discussion

This bicenter retrospective cohort study applied 1:1 propensity score matching to balance baseline confounding factors, systematically comparing the efficacy and safety of first-line third-generation EGFR-TKI plus pemetrexed-platinum chemotherapy *versus* third-generation EGFR-TKI monotherapy in patients with EGFR-sensitizing mutant lung adenocarcinoma and synchronous brain parenchymal metastases. Our results demonstrate that combination therapy improves intracranial and systemic objective response rates and prolongs iPFS, systemic PFS and OS relative to TKI monotherapy, with consistent survival benefits across clinical subgroups. Toxicities associated with the combination regimen were dominated by chemotherapy-related hematological and gastrointestinal adverse events and remained clinically manageable. Multivariate regression further confirmed that combination therapy constitutes an independent favorable prognostic factor for iPFS and OS.

Management of brain metastases remains a critical clinical challenge for patients with EGFR-mutant NSCLC. Widespread adoption of third-generation EGFR-TKIs has substantially improved intracranial disease control, yet acquired resistance and intracranial progression persist as major causes of treatment failure (Passaro et al., 2021). Strategies combining EGFR-TKI with chemotherapy have been well validated in the first-generation TKI era. The landmark phase III NEJ009 trial confirmed that gefitinib plus pemetrexed-carboplatin prolonged PFS and OS *versus* gefitinib monotherapy in the overall population, with consistent survival trends observed in patients with brain metastases (Miyauchi et al., 2022). The phase III GAP BRAIN trial, specifically focusing on patients with brain metastases, further demonstrated that gefitinib combined with chemotherapy extended median iPFS from 9.1 months to 15.2 months and reduced intracranial progression risk by 64%, verifying the intracranial therapeutic value of first-generation TKI plus chemotherapy (Hou et al., 2023). Multiple recent systematic reviews and network meta-analyses using indirect comparisons have suggested that third-generation EGFR-TKI combined with chemotherapy delivers optimal PFS and OS among all first-line regimens for EGFR-mutant NSCLC with brain metastases. However, such conclusions primarily rely on exploratory trial subgroup data, lacking large-scale real-world evidence dedicated to patients with brain parenchymal metastases (Ma et al., 2024; Chen et al., 2024).

In the era of third-generation TKIs, the FLAURA2 trial was the first phase III randomized controlled study to establish that osimertinib plus pemetrexed-platinum chemotherapy improved PFS and OS *versus* osimertinib monotherapy for unselected patients. Its predefined exploratory subgroup of 222 patients with baseline CNS metastases reported a 42% reduction in intracranial progression risk, comparable to the 46.4% risk reduction observed in our study (Planchard et al., 2023; Jänne et al., 2026). Notably, all patients enrolled in our cohort had confirmed brain parenchymal metastases, with isolated leptomeningeal metastasis excluded, yielding higher population homogeneity. Furthermore, our study incorporated multiple routinely administered third-generation TKIs including osimertinib, aumolertinib and furmonertinib, better reflecting real-world clinical practice. Mechanistically, brain metastatic lesions disrupt blood-brain barrier integrity, enabling pemetrexed penetration into intracranial tumor tissue to exert cytotoxic effects. In parallel, chemotherapy and EGFR-TKIs target distinct tumor cell subpopulations, synergistically eliminating resistant clones and inhibiting the proliferation of subclinical micrometastases, thereby delaying intracranial disease progression (Wang et al., 2025; Ruíz-Patiño et al., 2025).

Although the overall distribution of first intracranial progression patterns did not differ statistically between groups, the combination group exhibited numerically fewer new brain metastatic lesions. This observation suggests that the addition of chemotherapy exerts stronger inhibitory activity against intracranial subclinical micrometastases and reduces the emergence of new metastatic foci. This trend aligns with findings from the FLAURA trial, wherein osimertinib reduced new brain metastases compared with first-generation TKIs (Soria et al., 2018). Collectively, these data indicate that combining chemotherapy with third-generation TKIs strengthens comprehensive intracranial disease control and lowers the risk of *de novo* brain metastases. From the perspective of tumor clonal evolution, intracranial metastases display profound molecular heterogeneity. Partial micrometastatic lesions may exhibit low EGFR pathway dependence or bypass signaling activation, rendering single-agent TKI insufficient for eradication. Cytotoxic chemotherapy targets proliferative tumor cells independent of EGFR signaling status, enabling more comprehensive suppression of intracranial micrometastases (Ruíz-Patiño et al., 2025; Popat et al., 2023).

Safety considerations are central to clinical decision-making regarding combination regimens. In our analysis, the combination group demonstrated higher frequencies of any-grade and ≥grade 3 TRAEs than the monotherapy group. Incremental toxicities mainly comprised chemotherapy-associated neutropenia and nausea/vomiting, consistent with safety profiles documented in prior trials evaluating third-generation EGFR-TKI plus chemotherapy (Planchard et al., 2023). The incidence of ≥grade 3 neutropenia reached 15.0% in our cohort; all events resolved with supportive management without complicated febrile neutropenia. Gastrointestinal toxicities were predominantly grade 1–2 and effectively controlled by routine antiemetic prophylaxis. Critically, rates of CNS-specific adverse events (worsening peritumoral edema, treatment-related headache) were similar between groups, and no treatment-related intracranial hemorrhage, seizure or CNS death occurred across the entire cohort. These findings confirm that adding chemotherapy does not elevate the risk of intracranial-specific toxicity. Overall, toxicities of the combination regimen are predictable and clinically manageable without unexpected safety signals, consistent with expected tolerability in routine practice.

Our multivariate prognostic analyses revealed that beyond combination therapy, ECOG PS 0–1 and 1–3 brain metastases represented independent protective factors for iPFS, while symptomatic brain metastasis constituted a shared adverse prognostic factor for iPFS and OS. These results corroborate previous investigations into brain metastasis prognosis in lung cancer (Wang et al., 2025; Sereno et al., 2024), validating the utility of performance status, brain metastatic burden and symptomatic status for risk stratification. Importantly, combination therapy remained significantly associated with superior iPFS and OS after adjusting for these established prognostic markers, demonstrating that its survival benefit is independent of baseline clinical characteristics and represents a modifiable intervention to improve outcomes for patients with brain metastases. Additionally, extracranial metastatic burden independently predicted OS, highlighting the importance of systemic tumor control for long-term survival. By simultaneously enhancing intracranial and systemic disease control, combination therapy ultimately translates into improved overall survival.

The strengths of this study are multifaceted. First, we specifically targeted patients with EGFR-mutant lung adenocarcinoma and synchronous brain parenchymal metastases, representing one of the existing real-world studies comparing third-generation TKI plus chemotherapy *versus* monotherapy in this precise population. This work addresses limitations of phase III trials with small exploratory brain metastasis subgroups and suboptimal evidence certainty. Second, propensity score matching was implemented to balance baseline covariates and mitigate selection bias inherent to retrospective research; satisfactory post-matching covariate balance ensures reliable comparative results. Third, comprehensive endpoints were predefined, covering intracranial and systemic tumor responses, survival outcomes, patterns of intracranial progression, subgroup efficacy, and independent prognostic factors, providing multidimensional evidence to inform clinical decisions. Fourth, inclusion of multiple commonly used third-generation EGFR-TKIs enhances generalizability to routine clinical practice.

Several limitations should be acknowledged. First, as a retrospective observational study, even with PSM adjustment for measurable confounders, unmeasured confounding cannot be fully eliminated; the level of evidence remains inferior to prospective randomized controlled trials. Specifically, the post-matching SMD for symptomatic brain metastasis was 0.108, marginally above 0.1, with a slightly lower prevalence in the combination group (25.0% vs. 30.0%, P = 0.540). Because symptomatic brain metastasis was an independent adverse prognostic factor, this minor imbalance could, in theory, confer a modest baseline advantage on the combination arm and slightly overestimate its benefit; however, the absolute gap was only 5.0 percentage points, symptomatic status was adjusted for in all multivariable Cox models (after which combination therapy remained independently protective), and treatment benefit was consistent across symptomatic and asymptomatic subgroups (all interaction P > 0.05), suggesting a limited impact on our conclusions. Second, the moderate overall sample size yields limited event numbers within certain subgroups, reducing statistical power for subgroup analyses. No distinct population deriving preferential benefit was identified, and further validation using larger cohorts is required. Third, chemotherapy cycle counts, platinum agent selection, and duration of maintenance therapy were individualized by treating clinicians, introducing heterogeneity in treatment delivery that may influence outcomes. Fourth, only patients with brain parenchymal metastases were enrolled; conclusions cannot be directly extrapolated to individuals with isolated leptomeningeal metastases. Fifth, systematic profiling of molecular resistance mechanisms following combination therapy was not performed; the resistance landscape and optimal subsequent treatment strategies after combination therapy warrant further exploration. Sixth, complete data on post-progression (subsequent-line) therapy were not uniformly captured across the two centers, precluding a formal between-group comparison of subsequent regimens. Because later-line treatment can confound overall survival in retrospective cohorts, its potential influence warrants careful consideration. iPFS and systemic PFS terminate at the first documented progression and are therefore intrinsically unaffected by subsequent therapy, and both endpoints consistently favored the combination arm. Moreover, patients in the monotherapy group progressed earlier and thus had a longer post-progression window in which to receive salvage treatment (e.g., alternative-generation EGFR-TKIs, chemotherapy, anti-angiogenic agents, or intracranial radiotherapy); more intensive salvage care in this group would improve its post-progression survival and attenuate, rather than inflate, the combination arm’s OS advantage, thereby biasing the comparison toward the null. Nevertheless, residual confounding by subsequent therapy cannot be fully excluded without complete data, and our OS findings should accordingly be interpreted as associational rather than definitively causal; future studies with standardized capture of post-progression treatment and time-dependent covariate analyses are warranted. Finally, despite the median follow-up duration of 32.4 months, a subset of patients remain under surveillance. Extended follow-up will help definitively characterize the long-term survival advantage of the combination regimen.

## 5 Conclusion

In conclusion, our findings demonstrate that first-line third-generation EGFR-TKI combined with pemetrexed-platinum chemotherapy achieves significantly higher intracranial and systemic tumor response rates and prolongs iPFS, systemic PFS and OS compared with TKI monotherapy for patients with advanced EGFR-sensitizing mutant lung adenocarcinoma and synchronous brain parenchymal metastases, with acceptable safety and tolerability. This real-world study provides supportive evidence for clinical decision-making and favors the use of this combination regimen as a preferred first-line strategy for EGFR-mutant lung adenocarcinoma complicated by brain parenchymal metastases.

## Data Availability

The original contributions presented in the study are included in the article/Supplementary Material, further inquiries can be directed to the corresponding author.

## Glossary

AE: Adverse Event
ALK: Anaplastic Lymphoma Kinase
AUC: Area Under the Curve
CI: Confidence Interval
CNS: Central Nervous System
CR: Complete Response
CSCO: Chinese Society of Clinical Oncology
CT: Computed Tomography
CTCAE: National Cancer Institute Common Terminology Criteria for Adverse Events
DCR: Disease Control Rate
ECOG PS: Eastern Cooperative Oncology Group Performance Status
EGFR: Epidermal Growth Factor Receptor
GPA: Graded Prognostic Assessment
HR: Hazard Ratio
iDCR: Intracranial Disease Control Rate
iORR: Intracranial Objective Response Rate
iPFS: Intracranial Progression-Free Survival
IQR: Interquartile Range
MRI: Magnetic Resonance Imaging
NCCN: National Comprehensive Cancer Network
NSCLC: Non-Small Cell Lung Cancer
ORR: Objective Response Rate
OS: Overall Survival
PD: Progressive Disease
PET-CT: Positron Emission Tomography-Computed Tomography
PFS: Progression-Free Survival
PH: Proportional Hazards
PR: Partial Response
PSM: Propensity Score Matching
RECIST: Response Evaluation Criteria in Solid Tumors
ROS1: ROS Proto-Oncogene 1
SD: Stable Disease
SMD: Standardized Mean Difference
TKI: Tyrosine Kinase Inhibitor
TRAE: Treatment-Related Adverse Event
