# Supplementary material for: Third-generation EGFR-TKI plus pemetrexed-platinum chemotherapy versus TKI monotherapy as first-line treatment for EGFR-mutant lung adenocarcinoma with brain parenchymal metastases
Source: Front Pharmacol. 2026 Oct 7;17:1959801. doi: 10.3389/fphar.2026.1959801 (PMC13683676; doi:10.3389/fphar.2026.1959801)
Supplement: Supplementary file 1 [file Table1.docx]

****Supplementary Table S1.**** Scaled Schoenfeld residual (Grambsch–Therneau) tests of the proportional hazards assumption for the multivariate Cox models.

| **Variable** | **iPFS χ²** | **iPFS P** | **PFS χ²** | **PFS P** | **OS χ²** | **OS P** |
| --- | --- | --- | --- | --- | --- | --- |
| Treatment regimen | 0.71 | 0.399 | 0.95 | 0.330 | 0.58 | 0.446 |
| Age | 0.22 | 0.639 | 0.31 | 0.578 | 0.47 | 0.493 |
| ECOG PS | 1.46 | 0.227 | 1.18 | 0.277 | 1.73 | 0.188 |
| EGFR mutation subtype | 0.54 | 0.462 | 0.66 | 0.417 | 0.39 | 0.532 |
| Number of brain metastases | 1.82 | 0.177 | 1.51 | 0.219 | 1.04 | 0.308 |
| Symptomatic brain metastasis | 1.34 | 0.247 | 1.67 | 0.196 | 2.05 | 0.152 |
| No. of extracranial metastatic sites | 0.88 | 0.348 | 1.29 | 0.256 | 1.61 | 0.205 |
| **GLOBAL TEST (df=7)** | **6.93** | **0.436** | **7.58** | **0.371** | **8.21** | **0.314** |
